# Pediatric COVID-19: Immunopathogenesis, Transmission and Prevention

**DOI:** 10.3390/vaccines9091002

**Published:** 2021-09-08

**Authors:** Geraldine Blanchard-Rohner, Arnaud Didierlaurent, Anne Tilmanne, Pierre Smeesters, Arnaud Marchant

**Affiliations:** 1 Center of Vaccinology, Department of Pathology and Immunology, Faculty of Medicine, University of Geneva, 1205 Geneva, Switzerland; 2 Pediatric Immunology and Vaccinology Unit, Division of General Pediatrics, Department of Pediatrics, Gynecology and Obstetrics, Geneva University Hospitals, University of Geneva, 1205 Geneva, Switzerland; arnaud.didierlaurent@unige.ch; 3 Children’s Hospital of Geneva, 6, Rue Willy-Donzé, 1211 Geneva, Switzerland; 4 Children’s Hospital Queen Fabiola, Université libre de Bruxelles, 1020 Brussels, Belgium; anne.tilmanne@huderf.be (A.T.); pierre.smeesters@huderf.be (P.S.); 5 Institute for Medical Immunology, Université libre de Bruxelles, 1050 Charleroi, Belgium; Arnaud.Marchant@ulb.be

**Keywords:** COVID-19, SARS-CoV-2, pediatric, multisystem inflammatory syndrome in children (MIS-C), immunopathogenesis

## Abstract

Children are unique in the context of the COVID-19 pandemic. Overall, SARS-CoV-2 has a lower medical impact in children as compared to adults. A higher proportion of children than adults remain asymptomatic following SARS-CoV-2 infection and severe disease and death are also less common. This relative resistance contrasts with the high susceptibility of children to other respiratory tract infections. The mechanisms involved remain incompletely understood but could include the rapid development of a robust innate immune response. On the other hand, children develop a unique and severe complication, named multisystem inflammatory syndrome in children, several weeks after the onset of symptoms. Although children play an important role in the transmission of many pathogens, their contribution to the transmission of SARS-CoV-2 appears lower than that of adults. These unique aspects of COVID-19 in children must be considered in the benefit–risk analysis of vaccination. Several COVID-19 vaccines have been authorized for emergency use in adolescents and clinical studies are ongoing in children. As the vaccination of adolescents is rolled out in several countries, we shall learn about the impact of this strategy on the health of children and on transmission within communities.

## 1. Introduction

Since the beginning of the coronavirus disease 2019 (COVID-19) pandemic, children are underrepresented in terms of frequency and severity, accounting for less than 2% of diagnosed cases [1,2,3]. This under-representation is partly explained by the fact that children are less often diagnosed with a severe acute respiratory syndrome coronavirus 2 (SARS-CoV-2) infection, as they are less symptomatic than adults and because they appear less susceptible to the infection. 

It is estimated that up to 70% of children infected with SARS-CoV-2 remain asymptomatic [4,5,6]. In symptomatic children, clinical presentation is usually unspecific and indistinguishable from other respiratory virus infections, as the most frequent symptoms are fever and coughing, and up to 15% of children also present gastrointestinal symptoms, while anosmia is present in less than 1% of the cases [7].

If COVID-19 is a benign disease in most children, a very small proportion of pediatric patients develop severe disease and require hospitalization, accounting for only 1.5% of all COVID-19 hospital admissions [8]. Although it has been difficult to identify risk factors for severe disease in children, toddlers and adolescents are more likely to be hospitalized than young infants, and children with chronic pulmonary disease, congenital cardiac disease or neurological disease are more likely to be admitted to intensive care units [8]. The mortality associated with COVID-19 in children is very low, as pediatric deaths represent only 0.08% of all deaths associated with COVID-19 [9]. In the spring of 2020, clusters of children in Europe and America developed a severe hyperinflammatory syndrome resembling Kawasaki disease (KD) or toxic shock syndrome [10,11,12,13] several weeks after diagnosis of SARS-CoV-2 infection. The syndrome was named multisystem inflammatory syndrome in children (MIS-C) by the US Centers for Disease Control and the WHO, or paediatric inflammatory multisystem syndrome temporally associated with SARS-CoV-2 (PIMS-TS) by the UK Royal College of Paediatrics and Child Health (RCPCH).

It remains unclear why children are less susceptible to COVID-19 and why some develop MIS-C. Regarding the lower susceptibility, several hypotheses have been proposed, including cross-reactive immunity against seasonal coronaviruses to which children have been exposed and a lower expression of the angiotensin-converting enzyme 2 (ACE2) receptor, required for virus entry into human cells through interaction with the protein S, but data supporting these possibilities remain inconclusive. Data are emerging which suggest that children may develop a more rapid and more regulated immune response to SARS-CoV-2, allowing viral control with limited inflammation. This peculiar immune response profile could involve training of innate immunity by exposure to vaccines and pathogens in childhood [14]. Although children are less affected by COVID-19, they are infected and contribute to the transmission of SARS-CoV-2 [8]. Characterizing the immune response to SARS-CoV-2 in children who present with uncomplicated COVID-19 is required to understand the mechanisms underlying MIS-C and to define the immunological mechanisms controlling viral excretion and transmission that can be targeted by vaccination.

## 2. Immune Response to SARS-CoV-2 Infection in Children

Understanding why children are generally less prone to develop severe COVID-19 and associated symptoms could help to define immune mechanisms of protection against SARS-CoV-2 infection in the general population. The way children respond to SARS-CoV-2 is somewhat unusual, since the severity of infections with many other respiratory viruses, such as respiratory syncytial virus or influenza, is generally higher in children. This difference cannot be explained by a reduced viral load, as children have similar and sometimes higher viral copies in the first days of infection as compared to adults, but this viral load does not correlate with the severity of symptoms [15,16,17,18]. There is also no clear evidence that an age-dependent variation in the ACE2 expression level correlates with reduced disease severity. The gene expression of ACE2 in the nasal cavity and lungs was initially shown to be lower in young infants and to increase with age [17,19,20], but in later studies it was found to be similar in infected adults and children [21].

Because infection with common cold human coronaviruses (HCoVs)—HCoV-229E, -HKU1, -NL63 and -OC43—is frequent in children, it has been hypothesized that the presence of antibodies or cross-reactive T cells induced by HCoVs infection could provide protection in young individuals [22,23]. However, if children have high frequencies of class-switched B cells against SARS-CoV-2 and related coronaviruses, the level of HCoVs-specific antibodies is lower in children than in adults and was not associated with the risk of SARS-CoV-2 infection or MIS-C [21,24,25,26,27,28]. On the other hand, antibodies to HCoVs cross-react primarily with the S2 portion of the spike protein and, therefore, have limited capacity to neutralize SARS-CoV-2 [29]. It is possible that non-neutralizing HCoVs-specific antibodies could also contribute to viral control through Fc-dependent mechanisms and promote deleterious inflammatory responses, although evidence for a pathogenic role of antibodies in SARS-CoV-2 infection remains inconclusive [30,31,32,33]. Overall, the role of pre-existing and potentially cross-reactive HCoVs-specific antibodies in age-dependent susceptibility to SARS-CoV-2, or in disease severity, remains uncertain [34].

Early control of SARS-CoV-2 replication during primary infection is mainly mediated by the innate immune system [35]. Severe disease is associated with a lower initial interferon (IFN) response, followed by uncontrolled and persistent inflammation [36]. A key question is, therefore, whether children mount a less intense inflammatory response resulting in fewer and milder symptoms, or whether they have a more potent innate immune response, controlling viral replication more efficiently. Baseline cytokine levels are generally lower in children [37] and lower levels of inflammatory cytokines are detected in the lungs of children suffering from acute respiratory distress syndrome than in adults [38]. Studies initially reported that clinical parameters of inflammation were either undetectable or low in children with COVID-19 [14,39,40,41]. However, similar or higher systemic levels of cytokine were observed in later studies of hospitalized children and adults [21]. On the other hand, high levels of inflammatory cytokines were observed in severe cases of pediatric COVID-19 [42,43,44] and also in children with mild disease [28]. A pivotal study by Pierce et al. showed that children have a more intense nasal innate immune response, including higher levels of IFN-gamma and IFN-alpha, as compared to adults, suggesting that they may develop higher anti-viral responses at the mucosal level early on during infection [21]. A potent and efficient IFN response may, therefore, contribute to protection of children against COVID-19-associated symptoms [45]. A potential role for trained innate immunity, due to previous vaccinations or common infections, in the control of SARS-CoV-2 infection in children has been proposed [46] but currently lacks experimental evidence.

Analyses of peripheral blood immune cells in the first days after onset of symptoms revealed similar profiles in children and adults, including activation of monocytes and dendritic cells (DC) and transiently reduced numbers of lymphocytes, monocytes, DCs and NK cells [28,47]. Neutrophils appear to be less activated in pediatric, as compared to adult, COVID-19 cases, and this could mitigate tissue inflammation and damage [48]. On the other hand, children have higher numbers of circulating lymphocytes, which may contribute to better disease control [49,50,51]. As circulating T, B and NK cells decrease post-infection, they could be recruited at the site of infection earlier and in higher numbers in children than they are in adults.

Does a more efficient innate immune response result in a different adaptive immune response to SARS-CoV-2 in children, as compared to adults? It is now well established that children can mount a robust neutralizing antibody response to SARS-CoV-2 [17,52,53,54]. Initial reports from small pediatric cohort studies showed lower serum neutralizing activity, as compared to adults [21,55], and a reduced breadth of the antibody response to the spike protein [55]. A pre-print report indicates that the level of SARS-CoV-2 antibodies is reduced in children (*N* = 122) as compared to adults (*N* = 36), independently of disease severity, with a relatively higher proportion of antibodies targeting non-structural proteins, such as ORF3b and NSP1, than the nucleocapsid (N) or spike (S) proteins [56]. In contrast, other studies described similar levels of SARS-CoV-2 antibodies in both age groups, including those specific to the N protein [17,26,28,57,58]. Systems serology analyses, involving multiparametric assessment of antibody responses, showed a similar functional antibody profile, including phagocyte and complement-activating IgG, in children and adults with mild COVID-19 [33,54]. We recently conducted a longitudinal study of household contacts of COVID-19 cases and observed a similar magnitude and breadth of SARS-CoV-2-specific antibody response in children and adults with mild disease [28]. This study also indicated a more rapid onset of antibody response to the receptor-binding domain (RBD) and a more rapid appearance of peripheral blood B cell transcriptomic signature in children than in adults. A more rapid B cell response to SARS-CoV-2 in children could also contribute to a better control of the virus and to reduced symptoms. Indeed, levels of neutralizing antibodies and antibody-secreting B cells seven days after the onset of symptoms inversely correlated with viral load in children [59]. In addition, higher levels of somatic mutations in memory B cells and a more sustained antibody response were associated with a faster recovery from symptomatic COVID-19 in adults [60]. Limited data are available regarding the persistence of antibody responses in children. In a small cohort of children, anti-SARS-CoV-2 IgG declined six months after infection with lower levels than their infected parents at the same time point [61]. In a recent larger cohort study, children and adolescents showed high and durable antibody responses to SARS-CoV-2, following mild or asymptomatic infection [62]. Regarding cell-mediated immunity, only a few reports describe a lower or similar magnitude of SARS-CoV-2-specific T cells in children [21,57,63]. As the magnitude of the adaptive immune response to SARS-CoV-2 is related to severity of symptoms, it is essential that studies compare adults and children with similar clinical presentations.

## 3. Multisystem Inflammatory Syndrome in Children (MIS-C)

In contrast to children with acute COVID-19, children with MIS-C have no clinical features of active SARS-CoV-2 infection but have a history of COVID-19 or of contact with a person with COVID-19 around 4 weeks before the development of MIS-C symptoms [13]. Furthermore, MIS-C cases were typically observed 3–6 weeks following the peak incidence of COVID-19 in the general population [13,64]. Initially, MIS-C was reported as an atypical form of Kawasaki disease (KD) in regions of the world most affected by COVID-19, in Europe and in North and Latin America [11,12,65]. The exact incidence of MIS-C is uncertain. According to a study in New York, MIS-C occurs in two out of every 100,000 infected children [66]. Some ethnic groups were overrepresented, at least at the beginning of the pandemic, such as the African and Hispanic communities [10,11]. This could be explained by population-based genetic susceptibility; viral factors, as new variants of the virus might be more prone to induce immunopathological responses (for instance, due to epitopes with superantigen activity); or social determinants, as some ethnic minorities might have been more exposed to SARS-CoV-2 infection [67]. Children with MIS-C are older than children with KD, with a median age of 8–9 years, as compared to 3 years in KD [11,64].

Clinically, children with MIS-C present more heterogeneous symptoms than classical KD, as most children present with persistent fever, systemic inflammation, shock and multiple organ involvement [68]. Gastrointestinal manifestations are observed in 50–80% of MIS-C cases, sometimes with features of acute abdominal pain. They also frequently present with cutaneous rash and neurological symptoms, with features of meningitis and encephalitis [11,13,64,65,69,70]. Further, they very often present with cardiac dysfunction, with the occurrence of coronary artery dilation in 4–20% of children [11,64,71]. Patients often present with shock or hemodynamic instability; 60–80% require hospitalization in an intensive care unit; and 50% need inotropes and/or fluid resuscitation [11,64,72]. This syndrome usually resolves rapidly with corticosteroids and intravenous immunoglobulins (IVIG) [11,64,73,74,75]. The fatality rate is estimated to be 1–2% [66]. Inflammatory response in children with MIS-C is characterized by an increase in levels of C-reactive protein (CRP), procalcitonin, troponins, brain natriuretic peptide (BNP), ferritin and cytokines, such as IL-1, IL-6, IL-8, IL-10, IL-17, IL-18, IFN-γ and TNF, associated with profound lymphopenia and neutrophilia [10,11,13,74,76,77].

The physiopathology of MIS-C has been studied, but there is still no clear explanation as to why a small proportion of infected children develop MIS-C. It has been reported that the various symptoms of MIS-C reflect local vasculitis and inflammation of the affected organs. Similarly to KD, MIS-C is triggered by a previous infection and is likely an autoimmune syndrome. The inflammatory markers, such as CRP, ESR, procalcitonin and cytokines, appear more elevated in MIS-C as compared to other pediatric COVID-19 cases [13]. However, the inflammatory response is more intense in MIS-C compared to children with KD, and the cytokine profile is different; in KD, there is a robust IL17A increase, which is not observed in MIS-C. In contrast, MIS-C is associated with elevated levels of IL-1, which could be induced by endothelial cells damaged by autoantibodies and complement [78]. Autoantibodies of multiple specificities, including endothelial, gastrointestinal and immune cells, have indeed been observed in MIS-C [77,78]. These autoantibodies may form immune complexes and trigger immune damage to host tissues [77]. The production of autoantibodies may be due to cross-reactivity between SARS-CoV-2 and self-antigens. Although observed after infection, the production of autoantibodies has not been reported following COVID-19 vaccination (mainly targeting the spike antigen), suggesting that tissue damage due to SARS-CoV-2 infection involves other viral antigens than the S antigen. SARS-CoV-2 infection in the gastrointestinal tract may particularly favor the production of autoantibodies in MIS-C patients, and, in line with this hypothesis, many MIS-C cases have mesenteric adenitis and ileitis [11,65,79]. There are also possible roles played by direct virus dissemination and by a direct effect of the virus in the pathogenesis of MIS-C, as various autopsy reports have identified SARS-CoV-2 RNA on post-mortem tissues, in the extracellular compartment and within several cell types (cardiomyocytes, heart and brain endothelial cells, mesenchymal cells, macrophages and neutrophils) [80,81]. Therefore, the hyperinflammatory process and local vasculitis, combined with a direct, cytopathic effect of the virus in affected organs, could favor the onset of MIS-C. Another possibility is the occurrence of antibody-dependent enhancement (ADE) of coronavirus entry. Indeed, it has been suggested that SARS-CoV-2 antibodies bound to Fc receptors on macrophages and mast cells could favor virus entry and contribute to immune dysregulation [82,83]. The development of MIS-C may also involve a suppression of type I and type III interferon responses, which could first lead to an uncontrolled local viral replication with increased secretions of various cytokines, and then to an exaggerated adaptive immune response, involving B and T cells cross-reacting with self-antigens and triggering autoimmunity [78,84,85,86]. One hypothesis is that a unique part of the SARS-CoV-2 S protein could act as a superantigen, inducing oligoclonal activation of T cells [87]. This is supported by a recent report by Moreews et al., who observed an expansion of activated T cells expressing the Vbeta 21.3 T cell receptor beta-chain variable region in both CD4 and CD8 subsets in the majority of MIS-C patients, and not in control patients [88].

The humoral immune response in MIS-C patients appears to be different to that in children with uncomplicated COVID-19, although available data are limited. MIS-C patients produce neutralizing antibodies and have lower levels of IgM and higher levels of IgA and anti-spike IgG, as compared to other pediatric cases [89]. Another study reported that children with MIS-C had higher titers of SARS-CoV-2 spike RBD-specific IgG, as compared to children with uncomplicated COVID-19 [90]. All MIS-C children also had RBD IgM antibodies, suggesting recent SARS-CoV-2 infection. RBD IgG titers correlated with parameters of disease severity, such as erythrocyte sedimentation rate and duration of hospitalization and ICU stay [90]. These observations suggest that moderate levels of antibody protect against infection, while, above a certain threshold, higher levels of antibody may promote hyperinflammation.

It has been hypothesized that patients with MIS-C could have an unknown primary immunodeficiency [91]. Inborn defects of type I interferon responses are associated with severe COVID-19, with poor control of viral replication and excessive pulmonary and systemic inflammation [36,92,93]. Various immune defects have been associated with virus-triggered hyperinflammatory disease, such as herpes viruses and hemophagocytic lymphohistiocytosis (HLH). Several genes have been identified for HLH (such as PRF1, UNC13D, STX11 and STXBP2) that encode molecules involved in the cytotoxicity of CD8-T cells and NK cells [94]. In this case, CD8-T cells and NK cells cannot lyse infected cells and this results in excessive immune stimulation (43). Another example is XIAP or NLRC4 deficiencies, which are associated with an overstimulation of the inflammasome [95]. However, monogenic or oligogenic defects associated with MIS-C have not been identified yet.

## 4. SARS-CoV-2 Transmission by Children in Household Studies

Clinical clusters studies are a good way to understand the transmission of a disease. Many SARS-CoV-2 clusters have been reported and often include children, but rarely as the primary cases. The general consensus is that children are less frequently secondary cases than adults [96,97,98,99,100,101,102,103], or even that there is a similar attack rate across all age categories [104]. There are limited data about the infectivity of children showing a lower infectivity than adults. A Swiss study found that children were the first to develop SARS-CoV-2 symptoms in family clusters only in 8% (3/39) of the cases [105]. In 637 households in Israel, the infectivity of children was estimated to be 63% (95% CI: [37%, 88%]), relative to that of adults [106]. In South Korea, among 107 pediatric COVID-19 index cases and their 248 household members, the median age of pediatric COVID-19 index cases was 15 years (IQR 10–17 years), and the secondary attack rate was only 0.5% (95% CI 0.0–2.6%) [107].

Infectivity may vary with the age of children. A follow-up study of 5706 index cases in South Korea showed 11.8% (1248/10,592) and 1.9% (921/48,481) RT-PCR confirmed infection rates among household contacts and non-household contacts, respectively. The number of secondary cases was significantly different according to the age of the index case. For 0–9-year-old index cases, 5.3% of household contacts and 1.1% of non-household contacts were positive, whereas for 10–19-year-old index cases, 18.6% of household contacts and 0.9% of non-household contacts were positive, suggesting higher infectivity of older children [108]. These age-dependent transmission patterns, with higher secondary attack from adults (28.3%; 95% CI 20.2–37.1%) than from children (16.8%; 95% CI 12.3–21.7%; *p* < 0.001), have also been found in systematic reviews [109]. Outside the context of household transmission, data are scarce but confirm that children are rarely the primary source of secondary transmission in childcare and school settings and are more likely to be infected by an adult household member [110].

Clinical studies analyzing clusters of transmission, however, have several limitations, especially considering SARS-CoV-2 peculiarities. Children are often underrepresented as index cases, potentially because they are often asymptomatic [6,111]. However, the “index case” is often considered as the one infecting the rest of the family [112]. On the other hand, wide circulation of the virus among asymptomatic children has not been demonstrated [113]. It is, therefore, unlikely that children are the asymptomatic transmitters responsible for the infection of their relatives in the majority of familial clusters.

Important data were reported by Laxminarayan et al. [114]. Their tracing analysis of 84,965 confirmed cases and 575,071 contacts in two Indian provinces showed the highest probability of transmission within case–contact pairs of similar age. This enhanced transmission risk in similar age pairs seems strongest among children aged 0–14 years and among adults aged ≥65 years, although the greatest proportion of test-positive contacts within most age groups were exposed to index cases aged 20–44 years. An increased transmission risk within similar age pairs could be explained by different type and duration of contacts according to age, leading to a different risk of contamination. Such phenomena could contribute to the discrepancies seen in secondary attack rates across the studies, where attention is given to index cases or secondary infected people, but rarely to the transmitter–infected pair. However, cautions must be taken comparing epidemiological data over time, as shown by a study in the UK demonstrating differences between the two epidemiological waves. In contrast to the first COVID-19 wave, data suggested an increased risk of reported SARS-CoV-2 infection and COVID-19 among adults living with children during the second wave [115]. This difference could be explained by the different context in which the majority of children lived between the first and second waves—fewer contacts, due to fear of the virus and the closing of schools, during the first wave; and an increase in contacts, due to psycho-social necessity, less virus-related anxiety in the younger population and open schools, during the second wave.

Seroprevalence studies can also be used to estimate the incidence of SARS-CoV-2 infection. In Switzerland, a serosurvey showed a significantly lower seroprevalence among children aged 5–9 years (0.8%) and a lower risk of seropositivity compared to individuals aged 20–49 years (RR 0.32) [116], therefore confirming the studies on attack rate. Only few studies have been published on this topic, and it will also be important to assess the durability of antibodies following SARS-CoV-2 infection in children for the interpretation of seroprevalence studies [61,62].

## 5. Vaccination of Children

COVID-19 vaccination of children is the subject of several debates, and different approaches are currently being followed by countries where COVID-19 vaccines are sufficiently available to immunize this population. Clinical trials of mRNA and inactivated vaccines have been conducted in adolescents and have demonstrated a favorable safety profile and a similar immunogenicity to that observed in young adults [117,118]. Although sample size was relatively limited, as compared to adult studies, high efficacy of the BNT162b2 mRNA vaccine was shown in 12–15-year-old adolescents [117]. Immunogenicity and safety studies of mRNA vaccines are currently being conducted in younger children. Recent results showed immunogenicity of the Coronavac inactivated vaccine in children 3–11 years old [118]. Non-human primate studies showed potent and durable antibody and T cell responses to mRNA vaccines in infant animals [119]. Based on these studies, mRNA and inactivated COVID-19 vaccines have been approved for emergency use in adolescents by the regulatory authorities of several countries, including the US, Israel, the UK and China, and by the European Medicine Agency; vaccination of this population is being rolled out in an increasing number of countries. Notably, the UK recently decided to restrict vaccination to adolescents who are at risk of severe COVID-19 and to those who are household contacts of immunosuppressed patients (https://www.gov.uk/government/news/jcvi-issues-advice-on-COVID-19-vaccination-of-children-and-young-people, accessed on 31 July 2021) As for any vaccine in any target population, the decision to recommend and implement COVID-19 vaccination of adolescents and younger children is based on benefit–risk analyses [120,121,122,123]. Although most children remain asymptomatic, 6% of children are hospitalized; 13% of those hospitalized meet the criteria for severe disease with a fatality rate of 1%, while others suffer from prolonged symptoms (long COVID) and could, therefore, benefit from vaccination [124]. This benefit is stronger for vulnerable children who are at risk of severe COVID-19. On the other hand, although children are not predominant contributors to SARS-CoV-2 transmission, preventing infection by vaccination would indirectly reduce the risk of infection and disease for their contacts. This would be particularly important if those contacts were vulnerable and responded poorly to vaccination because of immunosuppression. Compulsory COVID-19 vaccination of children has been proposed to achieve high vaccination coverage, at the level of the population, and herd immunity [125,126,127]. However, as vaccine hesitancy in adults remains high in many countries, the prospect of reaching herd immunity, especially for variants of concern, appears more distant, and the argument of targeting children for compulsory vaccination has become weaker. Children would also benefit from COVID-19 vaccination if this would allow them to relax control measures, resulting in increased and diversified social contacts and reduced psychological impact of being a potential vector of the disease in the household [121]. Thus, several benefits of COVID-19 vaccination can be identified for healthy children, although globally they appear less marked than for adults. Although clinical trials have shown favorable safety profiles of mRNA and inactivated COVID-19 vaccines, the sample size of these studies is relatively limited, and long-term follow-up is still lacking. More safety data are being collected by countries where adolescent vaccination is being rolled out. This surveillance has allowed the identification of several cases of perimyocarditis following immunization of young adults and adolescents with the BNT162b2 or the mRNA-1273 vaccine, particularly in young male adults and adolescents [128,129,130]. These cases were rare, with an estimate of between 1/10,000 and 1/100,000 vaccinees, and were mild and self-limited. Therefore, it was concluded that the benefit of COVID-19 vaccination of adolescents, including the prevention of COVID-19 hospitalization, intensive care unit admission and death, outweighed the risk of perimyocarditis associated with mRNA vaccination [129]. As vaccine coverage of adults further increases, the proportion of COVID-19 cases in children will increase. With accumulating evidence of favorable safety profiles, COVID-19 vaccination of children is likely to be further promoted in the coming months and years.

## 6. Concluding Remarks

In contrast to respiratory tract infections caused by other viruses, SARS-CoV-2 has a lower impact on the health of children as compared to adults. However, severe disease, including MIS-C, has been observed and COVID-19 can be fatal in previously healthy children. Several hypotheses have been proposed to explain the favorable outcome of SARS-CoV-2 infection in children. Studies suggest that a robust, early innate immune response could play an important role. On the other hand, risk factors of severe COVID-19 and MIS-C remain poorly understood. Children at risk of severe COVID-19 because of underlying medical conditions can be offered vaccination. Identifying children at risk of MIS-C would help to extend this strategy. Based on their immunogenicity and safety profiles, several COVID-19 vaccines have been proposed to adolescents in an increasing number of countries. The lessons learned and experience gained from the COVID-19 pandemic will be very important for the prevention and the care of other infections affecting children and for the pathogens that will cause future pandemics.

## Data Availability

Not Applicable.

## References

[1] Wu Z., McGoogan J.M. (2020). Characteristics of and Important Lessons From the Coronavirus Disease 2019 (COVID-19) Outbreak in China. JAMA.

[2] Livingston E., Bucher K. (2020). Coronavirus Disease 2019 (COVID-19) in Italy. JAMA.

[3] CDC COVID-19 Response Team (2020). Coronavirus Disease 2019 in Children—United States, 12 February–2 April 2020. Morb. Mortal Wkly. Rep..

[4] Poline J., Gaschignard J., Leblanc C., Madhi F., Foucaud E., Nattes E., Faye A., Bonacorsi S., Mariani P., Varon E. (2020). Systematic SARS-CoV-2 Screening at Hospital Admission in Children: A French Prospective Multicenter Study. Clin. Infect Dis..

[5] Munro A.P.S., Faust S.N. (2020). COVID-19 in Children: Current Evidence and Key Questions. Curr. Opin. Infect Dis..

[6] Bellino S., Punzo O., Rota M.C., Manso M.D., Urdiales A.M., Andrianou X., Fabiani M., Boros S., Vescio F., Riccardo F. (2020). COVID-19 Disease Severity Risk Factors for Pediatric Patients in Italy. Pediatrics.

[7] Duarte-Salles T., Vizcaya D., Pistillo A., Casajust P., Sena A.G., Lai L.Y.H., Prats-Uribe A., Ahmed W.-U.-R., Alshammari T.M., Alghoul H. (2020). Baseline Characteristics, Management, and Outcomes of 55,270 Children and Adolescents Diagnosed with COVID-19 and 1,952,693 with Influenza in France, Germany, Spain, South Korea and the United States: An International Network Cohort Study. Medrxiv.

[8] Götzinger F., Santiago-García B., Noguera-Julián A., Lanaspa M., Lancella L., Carducci F.I.C., Gabrovska N., Velizarova S., Prunk P., Osterman V. (2020). COVID-19 in Children and Adolescents in Europe: A Multinational, Multicentre Cohort Study. Lancet Child Adolesc. Health.

[9] Bixler D., Miller A.D., Mattison C.P., Taylor B., Komatsu K., Pompa X.P., Moon S., Karmarkar E., Liu C.Y., Openshaw J.J. (2020). SARS-CoV-2–Associated Deaths Among Persons Aged <21 Years—United States, 12 February–31 July 2020. Mmwr. Morb. Mortal Wkly. Rep..

[10] Verdoni L., Mazza A., Gervasoni A., Martelli L., Ruggeri M., Ciuffreda M., Bonanomi E., D’Antiga L. (2020). An Outbreak of Severe Kawasaki-like Disease at the Italian Epicentre of the SARS-CoV-2 Epidemic: An Observational Cohort Study. Lancet.

[11] Whittaker E., Bamford A., Kenny J., Kaforou M., Jones C.E., Shah P., Ramnarayan P., Fraisse A., Miller O., Davies P. (2020). Clinical Characteristics of 58 Children with a Pediatric Inflammatory Multisystem Syndrome Temporally Associated with SARS-CoV-2. JAMA.

[12] Riphagen S., Gomez X., Gonzalez-Martinez C., Wilkinson N., Theocharis P. (2020). Hyperinflammatory Shock in Children during COVID-19 Pandemic. Lancet.

[13] Toubiana J., Poirault C., Corsia A., Bajolle F., Fourgeaud J., Angoulvant F., Debray A., Basmaci R., Salvador E., Biscardi S. (2020). Kawasaki-like Multisystem Inflammatory Syndrome in Children during the COVID-19 Pandemic in Paris, France: Prospective Observational Study. BMJ.

[14] Felsenstein S., Hedrich C.M. (2020). SARS-CoV-2 Infections in Children and Young People. Clin. Immunol..

[15] Aykac K., Yayla B.C.C., Ozsurekci Y., Evren K., Oygar P.D., Gurlevik S.L., Coskun T., Tasci O., Kaya F.D., Fidanci I. (2021). The Association of Viral Load and Disease Severity in Children with COVID-19. J. Med. Virol..

[16] Yonker L.M., Gilboa T., Ogata A.F., Senussi Y., Lazarovits R., Boribong B.P., Bartsch Y.C., Loiselle M., Rivas M.N., Porritt R.A. (2021). Multisystem Inflammatory Syndrome in Children Is Driven by Zonulin-Dependent Loss of Gut Mucosal Barrier. J. Clin. Investig..

[17] Yonker L.M., Neilan A.M., Bartsch Y., Patel A.B., Regan J., Arya P., Gootkind E., Park G., Hardcastle M., John A.S. (2020). Pediatric Severe Acute Respiratory Syndrome Coronavirus 2 (SARS-CoV-2): Clinical Presentation, Infectivity, and Immune Responses. J. Pediatrics.

[18] Heald-Sargent T., Muller W.J., Zheng X., Rippe J., Patel A.B., Kociolek L.K. (2020). Age-Related Differences in Nasopharyngeal Severe Acute Respiratory Syndrome Coronavirus 2 (SARS-CoV-2) Levels in Patients with Mild to Moderate Coronavirus Disease 2019 (COVID-19). JAMA Pediatr..

[19] Muus C., Luecken M.D., Eraslan G., Sikkema L., Waghray A., Heimberg G., Kobayashi Y., Vaishnav E.D., Subramanian A., Smillie C. (2021). Single-Cell Meta-Analysis of SARS-CoV-2 Entry Genes across Tissues and Demographics. Nat. Med..

[20] Bunyavanich S., Do A., Vicencio A. (2020). Nasal Gene Expression of Angiotensin-Converting Enzyme 2 in Children and Adults. JAMA.

[21] Pierce C.A., Sy S., Galen B., Goldstein D.Y., Orner E.P., Keller M.J., Herold K.C., Herold B.C. (2021). Natural Mucosal Barriers and COVID-19 in Children. JCI Insight.

[22] Steinman J.B., Lum F.M., Ho P.P.-K., Kaminski N., Steinman L. (2020). Reduced Development of COVID-19 in Children Reveals Molecular Checkpoints Gating Pathogenesis Illuminating Potential Therapeutics. Proc. Natl. Acad. Sci. USA.

[23] Ng K.W., Faulkner N., Cornish G.H., Rosa A., Harvey R., Hussain S., Ulferts R., Earl C., Wrobel A.G., Benton D.J. (2020). Preexisting and de Novo Humoral Immunity to SARS-CoV-2 in Humans. Science.

[24] Sermet-Gaudelus I., Temmam S., Huon C., Behillil S., Gajdos V., Bigot T., Lurier T., Chrétien D., Backovic M., Delaunay-Moisan A. (2021). Prior Infection by Seasonal Coronaviruses, as Assessed by Serology, Does Not Prevent SARS-CoV-2 Infection and Disease in Children, France, April to June 2020. Eurosurveillance.

[25] Gorse G.J., Donovan M.M., Patel G.B. (2020). Antibodies to Coronaviruses Are Higher in Older Compared with Younger Adults and Binding Antibodies Are More Sensitive than Neutralizing Antibodies in Identifying Coronavirus-associated Illnesses. J. Med. Virol..

[26] Selva K.J., van de Sandt C.E., Lemke M.M., Lee C.Y., Shoffner S.K., Chua B.Y., Davis S.K., Nguyen T.H.O., Rowntree L.C., Hensen L. (2021). Systems Serology Detects Functionally Distinct Coronavirus Antibody Features in Children and Elderly. Nat. Commun..

[27] Yang F., Nielsen S.C.A., Hoh R.A., Röltgen K., Wirz O.F., Haraguchi E., Jean G.H., Lee J.-Y., Pham T.D., Jackson K.J.L. (2021). Shared B Cell Memory to Coronaviruses and Other Pathogens Varies in Human Age Groups and Tissues. Science.

[28] Vono M., Huttner A., Lemeille S., Martinez-Murillo P., Meyer B., Baggio S., Sharma S., Thiriard A., Marchant A., Godeke G.-J. (2021). Robust Innate Responses to SARS-CoV-2 in Children Resolve Faster than in Adults without Compromising Adaptive Immunity. Cell Rep..

[29] Lv H., Wu N.C., Tsang O.T.-Y., Yuan M., Perera R.A.P.M., Leung W.S., So R.T.Y., Chan J.M.C., Yip G.K., Chik T.S.H. (2020). Cross-Reactive Antibody Response between SARS-CoV-2 and SARS-CoV Infections. Biorxiv.

[30] Arvin A.M., Fink K., Schmid M.A., Cathcart A., Spreafico R., Havenar-Daughton C., Lanzavecchia A., Corti D., Virgin H.W. (2020). A Perspective on Potential Antibody-Dependent Enhancement of SARS-CoV-2. Nature.

[31] Channappanavar R., Fehr A.R., Vijay R., Mack M., Zhao J., Meyerholz D.K., Perlman S. (2016). Dysregulated Type I Interferon and Inflammatory Monocyte-Macrophage Responses Cause Lethal Pneumonia in SARS-CoV-Infected Mice. Cell Host Microbe.

[32] Dandekar A.A., Perlman S. (2005). Immunopathogenesis of Coronavirus Infections: Implications for SARS. Nat. Rev. Immunol..

[33] Bartsch Y.C., Wang C., Zohar T., Fischinger S., Atyeo C., Burke J.S., Kang J., Edlow A.G., Fasano A., Baden L.R. (2021). Humoral Signatures of Protective and Pathological SARS-CoV-2 Infection in Children. Nat. Med..

[34] Anderson E.M., Goodwin E.C., Verma A., Arevalo C.P., Bolton M.J., Weirick M.E., Gouma S., McAllister C.M., Christensen S.R., Weaver J. (2021). Seasonal Human Coronavirus Antibodies Are Boosted upon SARS-CoV-2 Infection but Not Associated with Protection. Cell.

[35] Schultze J.L., Aschenbrenner A.C. (2021). COVID-19 and the Human Innate Immune System. Cell.

[36] Zhang Q., Bastard P., Liu Z., Pen J.L., Moncada-Velez M., Chen J., Ogishi M., Sabli I.K.D., Hodeib S., Korol C. (2020). Inborn Errors of Type I IFN Immunity in Patients with Life-Threatening COVID-19. Science.

[37] Decker M.-L., Gotta V., Wellmann S., Ritz N. (2017). Cytokine Profiling in Healthy Children Shows Association of Age with Cytokine Concentrations. Sci. Rep..

[38] Schouten L.R., van Kaam A.H., Kohse F., Veltkamp F., Bos L.D., de Beer F.M., van Hooijdonk R.T., Horn J., Straat M., Witteveen E. (2019). Age-Dependent Differences in Pulmonary Host Responses in ARDS: A Prospective Observational Cohort Study. Ann. Intensive Care.

[39] Henry B.M., Benoit S.W., de Oliveira M.H.S., Hsieh W.C., Benoit J., Ballout R.A., Plebani M., Lippi G. (2020). Laboratory Abnormalities in Children with Mild and Severe Coronavirus Disease 2019 (COVID-19): A Pooled Analysis and Review. Clin. Biochem..

[40] Moratto D., Giacomelli M., Chiarini M., Savarè L., Saccani B., Motta M., Timpano S., Poli P., Paghera S., Imberti L. (2020). Immune Response in Children with COVID-19 Is Characterized by Lower Levels of T-cell Activation than Infected Adults. Eur. J. Immunol..

[41] Lu X., Zhang L., Du H., Zhang J., Li Y.Y., Qu J., Zhang W., Wang Y., Bao S., Li Y. (2020). SARS-CoV-2 Infection in Children. N. Engl. J. Med..

[42] Vella L.A., Giles J.R., Baxter A.E., Oldridge D.A., Diorio C., Kuri-Cervantes L., Alanio C., Pampena M.B., Wu J.E., Chen Z. (2021). Deep Immune Profiling of MIS-C Demonstrates Marked but Transient Immune Activation Compared to Adult and Pediatric COVID-19. Sci. Immunol..

[43] Lu W., Yang L., Li X., Sun M., Zhang A., Qi S., Chen Z., Zhang L., Li J., Xiong H. (2021). Early Immune Responses and Prognostic Factors in Children with COVID-19: A Single-Center Retrospective Analysis. BMC Pediatr..

[44] Wu H., Zhu H., Yuan C., Yao C., Luo W., Shen X., Wang J., Shao J., Xiang Y. (2020). Clinical and Immune Features of Hospitalized Pediatric Patients With Coronavirus Disease 2019 (COVID-19) in Wuhan, China. JAMA Netw. Open.

[45] Lucas C., Wong P., Klein J., Castro T.B.R., Silva J., Sundaram M., Ellingson M.K., Mao T., Oh J.E., Israelow B. (2020). Longitudinal Analyses Reveal Immunological Misfiring in Severe COVID-19. Nature.

[46] Mantovani A., Rinaldi E., Zusi C., Beatrice G., Saccomani M.D., Dalbeni A. (2021). Coronavirus Disease 2019 (COVID-19) in Children and/or Adolescents: A Meta-Analysis. Pediatr. Res..

[47] Neeland M.R., Bannister S., Clifford V., Dohle K., Mulholland K., Sutton P., Curtis N., Steer A.C., Burgner D.P., Crawford N.W. (2021). Innate Cell Profiles during the Acute and Convalescent Phase of SARS-CoV-2 Infection in Children. Nat. Commun..

[48] Seery V., Raiden S.C., Algieri S.C., Grisolía N.A., Filippo D., Carli N.D., Lalla S.D., Cairoli H., Chiolo M.J., Meregalli C.N. (2021). Blood Neutrophils from Children with COVID-19 Exhibit Both Inflammatory and Anti-Inflammatory Markers. Ebiomedicine.

[49] VAKKILA J., THOMSON A.W., VETTENRANTA K., SARIOLA H., SAARINEN-PIHKALA U.M. (2004). Dendritic Cell Subsets in Childhood and in Children with Cancer: Relation to Age and Disease Prognosis. Clin. Exp. Immunol..

[50] Valiathan R., Ashman M., Asthana D. (2016). Effects of Ageing on the Immune System: Infants to Elderly. Scand. J. Immunol..

[51] Erkeller-Yuksel F.M., Deneys V., Yuksel B., Hannet I., Hulstaert F., Hamilton C., Mackinnon H., Stokes L.T., Munhyeshuli V., Vanlangendonck F. (1992). Age-Related Changes in Human Blood Lymphocyte Subpopulations. J. Pediatrics.

[52] Liu P., Cai J., Jia R., Xia S., Wang X., Cao L., Zeng M., Xu J. (2020). Dynamic Surveillance of SARS-CoV-2 Shedding and Neutralizing Antibody in Children with COVID-19. Emerg. Microbes Infec..

[53] Zhang Y., Xu J., Jia R., Yi C., Gu W., Liu P., Dong X., Zhou H., Shang B., Cheng S. (2020). Protective Humoral Immunity in SARS-CoV-2 Infected Pediatric Patients. Cell Mol. Immunol..

[54] Chakraborty S., Gonzalez J., Edwards K., Mallajosyula V., Buzzanco A.S., Sherwood R., Buffone C., Kathale N., Providenza S., Xie M.M. (2021). Proinflammatory IgG Fc Structures in Patients with Severe COVID-19. Nat. Immunol..

[55] Weisberg S.P., Connors T.J., Zhu Y., Baldwin M.R., Lin W.-H., Wontakal S., Szabo P.A., Wells S.B., Dogra P., Gray J. (2021). Distinct Antibody Responses to SARS-CoV-2 in Children and Adults across the COVID-19 Clinical Spectrum. Nat. Immunol..

[56] Hachim A., Gu H., Kavian O., Kwan M.Y., Chan W., Yau Y.S., Chiu S.S., Tsang O.T., Hui D.S., Ma F. (2021). The SARS-CoV-2 Antibody Landscape Is Lower in Magnitude for Structural Proteins, Diversified for Accessory Proteins and Stable Long-Term in Children. Medrxiv.

[57] Goenka A., Halliday A., Gregorova M., Milodowski E., Thomas A., Williamson M.K., Baum H., Oliver E., Long A.E., Knezevic L. (2021). Young Infants Exhibit Robust Functional Antibody Responses and Restrained IFN-γ Production to SARS-CoV-2. Cell Rep. Med..

[58] Tosif S., Neeland M.R., Sutton P., Licciardi P.V., Sarkar S., Selva K.J., Do L.A.H., Donato C., Toh Z.Q., Higgins R. (2020). Immune Responses to SARS-CoV-2 in Three Children of Parents with Symptomatic COVID-19. Nat. Commun..

[59] Cotugno N., Ruggiero A., Bonfante F., Petrara M.R., Zicari S., Pascucci G.R., Zangari P., Ioris M.A.D., Santilli V., Manno E.C. (2021). Virological and Immunological Features of SARS-CoV-2-Infected Children Who Develop Neutralizing Antibodies. Cell Rep..

[60] Chen Y., Zuiani A., Fischinger S., Mullur J., Atyeo C., Travers M., Lelis F.J.N., Pullen K.M., Martin H., Tong P. (2020). Quick COVID-19 Healers Sustain Anti-SARS-CoV-2 Antibody Production. Cell.

[61] Bloise S., Marcellino A., Testa A., Dilillo A., Mallardo S., Isoldi S., Martucci V., Sanseviero M.T., Giudice E.D., Iorfida D. (2021). Serum IgG Levels in Children 6 Months after SARS-CoV-2 Infection and Comparison with Adults. Eur. J. Pediatr..

[62] Garrido C., Hurst J.H., Lorang C.G., Aquino J.N., Rodriguez J., Pfeiffer T.S., Singh T., Semmes E.C., Lugo D.J., Rotta A.T. (2021). Asymptomatic or Mild Symptomatic SARS-CoV-2 Infection Elicits Durable Neutralizing Antibody Responses in Children and Adolescents. JCI Insight.

[63] Cohen C.A., Li A.P., Hachim A., Hui D.S., Kwan M.Y., Tsang O.T., Chiu S.S., Chan W.H., Yau Y.S., Kavian N. (2021). SARS-CoV-2 Specific T Cell Responses Are Lower in Children and Increase with Age and Time after Infection. Medrxiv.

[64] Feldstein L.R., Rose E.B., Horwitz S.M., Collins J.P., Newhams M.M., Son M.B.F., Newburger J.W., Kleinman L.C., Heidemann S.M., Martin A.A. (2020). Multisystem Inflammatory Syndrome in U.S. Children and Adolescents. N. Engl. J. Med..

[65] Grazioli S., Tavaglione F., Torriani G., Wagner N., Rohr M., L’Huillier A.G., Leclercq C., Perrin A., Bordessoule A., Beghetti M. (2020). Immunological Assessment of Pediatric Multisystem Inflammatory Syndrome Related to COVID-19. J. Pediatric Infect. Dis. Soc..

[66] Ahmed M., Advani S., Moreira A., Zoretic S., Martinez J., Chorath K., Acosta S., Naqvi R., Burmeister-Morton F., Burmeister F. (2020). Multisystem Inflammatory Syndrome in Children: A Systematic Review. Eclinicalmedicine.

[67] Cheng M.H., Zhang S., Porritt R.A., Arditi M., Bahar I. (2020). An Insertion Unique to SARS-CoV-2 Exhibits Superantigenic Character Strengthened by Recent Mutations. Biorxiv.

[68] Cattalini M., Paolera S.D., Zunica F., Bracaglia C., Giangreco M., Verdoni L., Meini A., Sottile R., Caorsi R., Zuccotti G. (2021). Defining Kawasaki Disease and Pediatric Inflammatory Multisystem Syndrome-Temporally Associated to SARS-CoV-2 Infection during SARS-CoV-2 Epidemic in Italy: Results from a National, Multicenter Survey. Pediatr. Rheumatol..

[69] Harwood R., Allin B., Jones C.E., Whittaker E., Ramnarayan P., Ramanan A.V., Kaleem M., Tulloh R., Peters M.J., Almond S. (2020). A National Consensus Management Pathway for Paediatric Inflammatory Multisystem Syndrome Temporally Associated with COVID-19 (PIMS-TS): Results of a National Delphi Process. Lancet Child. Adolesc. Health.

[70] Abrams J.Y., Godfred-Cato S.E., Oster M.E., Chow E.J., Koumans E.H., Bryant B., Leung J.W., Belay E.D. (2020). Multisystem Inflammatory Syndrome in Children Associated with Severe Acute Respiratory Syndrome Coronavirus 2: A Systematic Review. J. Pediatrics.

[71] Belhadjer Z., Méot M., Bajolle F., Khraiche D., Legendre A., Abakka S., Auriau J., Grimaud M., Oualha M., Beghetti M. (2020). Acute Heart Failure in Multisystem Inflammatory Syndrome in Children (MIS-C) in the Context of Global SARS-CoV-2 Pandemic. Circulation.

[72] Dufort E.M., Koumans E.H., Chow E.J., Rosenthal E.M., Muse A., Rowlands J., Barranco M.A., Maxted A.M., Rosenberg E.S., Easton D. (2020). Multisystem Inflammatory Syndrome in Children in New York State. N. Engl. J. Med..

[73] Ouldali N., Toubiana J., Antona D., Javouhey E., Madhi F., Lorrot M., Léger P.-L., Galeotti C., Claude C., Wiedemann A. (2021). Association of Intravenous Immunoglobulins Plus Methylprednisolone vs. Immunoglobulins Alone with Course of Fever in Multisystem Inflammatory Syndrome in Children. JAMA.

[74] Diorio C., Henrickson S.E., Vella L.A., McNerney K.O., Chase J.M., Burudpakdee C., Lee J.H., Jasen C., Balamuth F., Barrett D.M. (2020). Multisystem Inflammatory Syndrome in Children and COVID-19 Are Distinct Presentations of SARS-CoV-2. J. Clin. Investig..

[75] Henderson L.A., Canna S.W., Schulert G.S., Volpi S., Lee P.Y., Kernan K.F., Caricchio R., Mahmud S., Hazen M.M., Halyabar O. (2020). On the Alert for Cytokine Storm: Immunopathology in COVID-19. Arthritis Rheumatol..

[76] Carter M.J., Fish M., Jennings A., Doores K.J., Wellman P., Seow J., Acors S., Graham C., Timms E., Kenny J. (2020). Peripheral Immunophenotypes in Children with Multisystem Inflammatory Syndrome Associated with SARS-CoV-2 Infection. Nat. Med..

[77] Gruber C., Patel R.S., Trachtman R., Lepow L., Amanat F., Krammer F., Wilson K.M., Onel K., Geanon D., Tuballes K. (2020). Mapping Systemic Inflammation and Antibody Responses in Multisystem Inflammatory Syndrome in Children (MIS-C). Cell.

[78] Consiglio C.R., Cotugno N., Sardh F., Pou C., Amodio D., Rodriguez L., Tan Z., Zicari S., Ruggiero A., Pascucci G.R. (2020). The Immunology of Multisystem Inflammatory Syndrome in Children with COVID-19. Cell.

[79] Lamers M.M., Beumer J., van der Vaart J., Knoops K., Puschhof J., Breugem T.I., Ravelli R.B.G., van Schayck J.P., Mykytyn A.Z., Duimel H.Q. (2020). SARS-CoV-2 Productively Infects Human Gut Enterocytes. Biorxiv.

[80] Duarte-Neto A.N., Caldini E.G., Gomes-Gouvêa M.S., Kanamura C.T., de Almeida Monteiro R.A., Ferranti J.F., Ventura A.M.C., Regalio F.A., Fiorenzano D.M., Gibelli M.A.B.C. (2021). An Autopsy Study of the Spectrum of Severe COVID-19 in Children: From SARS to Different Phenotypes of MIS-C. Eclinicalmedicine.

[81] Dolhnikoff M., Ferranti J.F., de Almeida Monteiro R.A., Duarte-Neto A.N., Gomes-Gouvêa M.S., Degaspare N.V., Delgado A.F., Fiorita C.M., Leal G.N., Rodrigues R.M. (2020). SARS-CoV-2 in Cardiac Tissue of a Child with COVID-19-Related Multisystem Inflammatory Syndrome. Lancet Child. Adolesc. Health.

[82] Wan Y., Shang J., Sun S., Tai W., Chen J., Geng Q., He L., Chen Y., Wu J., Shi Z. (2020). Molecular Mechanism for Antibody-Dependent Enhancement of Coronavirus Entry. J. Virol..

[83] Ricke D.O. (2021). Two Different Antibody-Dependent Enhancement (ADE) Risks for SARS-CoV-2 Antibodies. Front. Immunol..

[84] Park A., Iwasaki A. (2020). Type I and Type III Interferons—Induction, Signaling, Evasion, and Application to Combat COVID-19. Cell Host Microbe.

[85] Blanco-Melo D., Nilsson-Payant B.E., Liu W.-C., Uhl S., Hoagland D., Møller R., Jordan T.X., Oishi K., Panis M., Sachs D. (2020). Imbalanced Host Response to SARS-CoV-2 Drives Development of COVID-19. Cell.

[86] Caldarale F., Giacomelli M., Garrafa E., Tamassia N., Morreale A., Poli P., Timpano S., Baresi G., Zunica F., Cattalini M. (2021). Plasmacytoid Dendritic Cells Depletion and Elevation of IFN-γ Dependent Chemokines CXCL9 and CXCL10 in Children With Multisystem Inflammatory Syndrome. Front. Immunol..

[87] Cheng M.H., Zhang S., Porritt R.A., Rivas M.N., Paschold L., Willscher E., Binder M., Arditi M., Bahar I. (2020). Superantigenic Character of an Insert Unique to SARS-CoV-2 Spike Supported by Skewed TCR Repertoire in Patients with Hyperinflammation. Proc. Natl. Acad. Sci. USA.

[88] Moreews M., Gouge K.L., Khaldi-Plassart S., Pescarmona R., Mathieu A.-L., Malcus C., Djebali S., Bellomo A., Dauwalder O., Perret M. (2021). Polyclonal Expansion of TCR Vbeta 21.3+ CD4+ and CD8+ T Cells Is a Hallmark of Multisystem Inflammatory Syndrome in Children. Sci. Immunol..

[89] Anderson E.M., Diorio C., Goodwin E.C., McNerney K.O., Weirick M.E., Gouma S., Bolton M.J., Arevalo C.P., Chase J., Hicks P. (2020). SARS-CoV-2 Antibody Responses in Children with MIS-C and Mild and Severe COVID-19. J. Pediatric Infect. Dis. Soc..

[90] Rostad C.A., Chahroudi A., Mantus G., Lapp S.A., Teherani M., Macoy L., Tarquinio K.M., Basu R.K., Kao C., Linam W.M. (2020). Quantitative SARS-CoV-2 Serology in Children With Multisystem Inflammatory Syndrome (MIS-C). Pediatrics.

[91] Casanova J.-L., Abel L. (2020). The Human Genetic Determinism of Life-Threatening Infectious Diseases: Genetic Heterogeneity and Physiological Homogeneity?. Hum. Genet..

[92] Hadjadj J., Yatim N., Barnabei L., Corneau A., Boussier J., Pere H., Charbit B., Bondet V., Chenevier-Gobeaux C., Breillat P. (2020). Impaired Type I Interferon Activity and Exacerbated Inflammatory Responses in Severe COVID-19 Patients. Medrxiv.

[93] Bastard P., Rosen L.B., Zhang Q., Michailidis E., Hoffmann H.-H., Zhang Y., Dorgham K., Philippot Q., Rosain J., Béziat V. (2020). Autoantibodies against Type I IFNs in Patients with Life-Threatening COVID-19. Science.

[94] Janka G.E. (2012). Familial and Acquired Hemophagocytic Lymphohistiocytosis. Annu. Rev. Med..

[95] Canna S.W., Marsh R.A. (2020). Pediatric Hemophagocytic Lymphohistiocytosis. Blood.

[96] Wang Z., Ma W., Zheng X., Wu G., Zhang R. (2020). Household Transmission of SARS-CoV-2. J. Infect..

[97] Li W., Zhang B., Lu J., Liu S., Chang Z., Cao P., Liu X., Zhang P., Ling Y., Tao K. (2020). The Characteristics of Household Transmission of COVID-19. Clin. Infect. Dis..

[98] Jing Q.-L., Liu M.-J., Zhang Z.-B., Fang L.-Q., Yuan J., Zhang A.-R., Dean N.E., Luo L., Ma M.-M., Longini I. (2020). Household Secondary Attack Rate of COVID-19 and Associated Determinants in Guangzhou, China: A Retrospective Cohort Study. Lancet Infect. Dis..

[99] Mizumoto K., Omori R., Nishiura H. (2020). Age Specificity of Cases and Attack Rate of Novel Coronavirus Disease (COVID-19). Medrxiv.

[100] Zhang J., Litvinova M., Liang Y., Wang Y., Wang W., Zhao S., Wu Q., Merler S., Viboud C., Vespignani A. (2020). Changes in Contact Patterns Shape the Dynamics of the COVID-19 Outbreak in China. Science.

[101] Reukers D.F.M., van Boven M., Meijer A., Rots N., Reusken C., Roof I., van Gageldonk-Lafeber A.B., van der Hoek W., van den Hof S. (2021). High Infection Secondary Attack Rates of SARS-CoV-2 in Dutch Households Revealed by Dense Sampling. Clin. Infect. Dis..

[102] Davies N.G., Klepac P., Liu Y., Prem K., Jit M., Eggo R.M., CMMID COVID-19 working group (2020). Age-Dependent Effects in the Transmission and Control of COVID-19 Epidemics. Nat. Med..

[103] Koh W.C., Naing L., Chaw L., Rosledzana M.A., Alikhan M.F., Jamaludin S.A., Amin F., Omar A., Shazli A., Griffith M. (2020). What Do We Know about SARS-CoV-2 Transmission? A Systematic Review and Meta-Analysis of the Secondary Attack Rate and Associated Risk Factors. PLoS ONE.

[104] Bi Q., Wu Y., Mei S., Ye C., Zou X., Zhang Z., Liu X., Wei L., Truelove S.A., Zhang T. (2020). Epidemiology and Transmission of COVID-19 in 391 Cases and 1286 of Their Close Contacts in Shenzhen, China: A Retrospective Cohort Study. Lancet Infect. Dis..

[105] Posfay-Barbe K.M., Wagner N., Gauthey M., Moussaoui D., Loevy N., Diana A., L’Huillier A.G. (2020). COVID-19 in Children and the Dynamics of Infection in Families. Pediatrics.

[106] Dattner I., Goldberg Y., Katriel G., Yaari R., Gal N., Miron Y., Ziv A., Sheffer R., Hamo Y., Huppert A. (2021). The Role of Children in the Spread of COVID-19: Using Household Data from Bnei Brak, Israel, to Estimate the Relative Susceptibility and Infectivity of Children. PLoS Comput. Biol..

[107] Kim J., Choe Y.J., Lee J., Park Y.J., Park O., Han M.S., Kim J.-H., Choi E.H. (2021). Role of Children in Household Transmission of COVID-19. Arch. Dis. Child..

[108] Park Y.J., Choe Y.J., Park O., Park S.Y., Kim Y.-M., Kim J., Kweon S., Woo Y., Gwack J., Kim S.S. (2020). Contact Tracing during Coronavirus Disease Outbreak, South Korea, 2020—Volume 26, Number 10—October 2020—Emerging Infectious Diseases Journal—CDC. Emerg. Infect. Dis..

[109] Madewell Z.J., Yang Y., Longini I.M., Halloran M.E., Dean N.E. (2020). Household Transmission of SARS-CoV-2. JAMA Netw. Open.

[110] Zimmerman K.O., Akinboyo I.C., Brookhart M.A., Boutzoukas A.E., McGann K.A., Smith M.J., Panayotti G.M., Armstrong S.C., Bristow H., Parker D. (2021). Incidence and Secondary Transmission of SARS-CoV-2 Infections in Schools. Pediatrics.

[111] Zhu Y., Bloxham C.J., Hulme K.D., Sinclair J.E., Tong Z.W.M., Steele L.E., Noye E.C., Lu J., Xia Y., Chew K.Y. (2020). A Meta-Analysis on the Role of Children in SARS-CoV-2 in Household Transmission Clusters. Clin. Infect. Dis..

[112] Liu Z., Chu R., Gong L., Su B., Wu J. (2020). The Assessment of Transmission Efficiency and Latent Infection Period in Asymptomatic Carriers of SARS-CoV-2 Infection. Int. J. Infect. Dis..

[113] Ulyte A., Radtke T., Abela I.A., Haile S.R., Berger C., Huber M., Schanz M., Schwarzmueller M., Trkola A., Fehr J. (2021). Clustering and Longitudinal Change in SARS-CoV-2 Seroprevalence in School Children in the Canton of Zurich, Switzerland: Prospective Cohort Study of 55 Schools. BMJ.

[114] Laxminarayan R., Wahl B., Dudala S.R., Gopal K., B C.M., Neelima S., Reddy K.S.J., Radhakrishnan J., Lewnard J.A. (2020). Epidemiology and Transmission Dynamics of COVID-19 in Two Indian States. Science.

[115] Forbes H., Morton C.E., Bacon S., McDonald H.I., Minassian C., Brown J.P., Rentsch C.T., Mathur R., Schultze A., DeVito N.J. (2021). Association between Living with Children and Outcomes from COVID-19: OpenSAFELY Cohort Study of 12 Million Adults in England. BMJ.

[116] Stringhini S., Wisniak A., Piumatti G., Azman A.S., Lauer S.A., Baysson H., Ridder D.D., Petrovic D., Schrempft S., Marcus K. (2020). Seroprevalence of Anti-SARS-CoV-2 IgG Antibodies in Geneva, Switzerland (SEROCoV-POP): A Population-Based Study. Lancet.

[117] Frenck R.W., Klein N.P., Kitchin N., Gurtman A., Absalon J., Lockhart S., Perez J.L., Walter E.B., Senders S., Bailey R. (2021). Safety, Immunogenicity, and Efficacy of the BNT162b2 COVID-19 Vaccine in Adolescents. N. Engl. J. Med..

[118] Han B., Song Y., Li C., Yang W., Ma Q., Jiang Z., Li M., Lian X., Jiao W., Wang L. (2021). Safety, Tolerability, and Immunogenicity of an Inactivated SARS-CoV-2 Vaccine (CoronaVac) in Healthy Children and Adolescents: A Double-Blind, Randomised, Controlled, Phase 1/2 Clinical Trial. Lancet Infect. Dis..

[119] Garrido C., Curtis A.D., Dennis M., Pathak S.H., Gao H., Montefiori D., Tomai M., Fox C.B., Kozlowski P.A., Scobey T. (2021). SARS-CoV-2 Vaccines Elicit Durable Immune Responses in Infant Rhesus Macaques. Sci. Immunol..

[120] Li G., Finn A., Pollard A.J. (2021). Should We Be Vaccinating Children against COVID-19 in High-Income Countries?. Expert Rev. Vaccines.

[121] Frenck R.W., Klein N.P., Kitchin N., Gurtman A., Absalon J., Lockhart S., Perez J.L., Walter E.B., Senders S., Bailey R. (2021). Education and Mental Health: Good Reasons to Vaccinate Children. Lancet.

[122] Callaway E. (2021). COVID Vaccines and Kids: Five Questions as Trials Begin. Nature.

[123] Ladhani S.N. (2021). Crossing the Rubicon: A Fine Line between Waiting and Vaccinating Adolescents against COVID-19. J. Infect..

[124] Martin B., DeWitt P.E., Russell S., Anand A., Bradwell K.R., Bremer C., Gabriel D., Girvin A.T., Hajagos J.G., McMurry J.A. (2021). Children with SARS-CoV-2 in the National COVID Cohort Collaborative (N3C). Medrxiv.

[125] Plotkin S.A., Levy O. (2021). Considering Mandatory Vaccination of Children for COVID-19. Pediatrics.

[126] Archard D., Brierley J., Cave E. (2021). Compulsory Childhood Vaccination: Human Rights, Solidarity, and Best Interests. Med. Law Rev..

[127] Gostin L.O., Shaw J., Salmon D.A. (2021). Mandatory SARS-CoV-2 Vaccinations in K-12 Schools, Colleges/Universities, and Businesses. JAMA.

[128] Jhaveri R., Adler-Shohet F.C., Blyth C.C., Chiotos K., Gerber J.S., Green M., Kociolek L., Martin-Blais R., Palazzi D., Shane A.L. (2021). Weighing the Risks of Perimyocarditis With the Benefits of SARS-CoV-2 MRNA Vaccination in Adolescents. J. Pediatric Infect. Dis. Soc..

[129] Gargano J.W., Wallace M., Hadler S.C., Langley G., Su J.R., Oster M.E., Broder K.R., Gee J., Weintraub E., Shimabukuro T. (2021). Use of MRNA COVID-19 Vaccine After Reports of Myocarditis Among Vaccine Recipients: Update from the Advisory Committee on Immunization Practices—United States, June 2021. Mmwr Morb. Mortal Wkly. Rep..

[130] Schauer J., Buddhe S., Colyer J., Sagiv E., Law Y., Chikkabyrappa S.M., Portman M.A. (2021). Myopericarditis after the Pfizer MRNA COVID-19 Vaccine in Adolescents. J. Pediatrics.

